# Towards Wearable Augmented Reality in Healthcare: A Comparative Survey and Analysis of Head-Mounted Displays

**DOI:** 10.3390/ijerph20053940

**Published:** 2023-02-22

**Authors:** Yahia Baashar, Gamal Alkawsi, Wan Nooraishya Wan Ahmad, Mohammad Ahmed Alomari, Hitham Alhussian, Sieh Kiong Tiong

**Affiliations:** 1 Faculty of Computing and Informatics, Universiti Malaysia Sabah (UMS), Labuan 87000, Malaysia; 2 Institute of Sustainable Energy (ISE), Universiti Tenaga Nasional, Kajang 43000, Malaysia; 3 Faculty of Computer Science and Information Systems, Thamar University, Thamar 87246, Yemen; 4 Institute of Informatics and Computing in Energy, Universiti Tenaga Nasional (UNITEN), Kajang 43000, Malaysia; 5 Department of Computer and Information Sciences, Universiti Teknologi PETRONAS, Seri Iskandar 32610, Malaysia

**Keywords:** head-mounted display, smart glasses, Google Glass, Microsoft HoloLens, healthcare, clinical settings

## Abstract

Head-mounted displays (HMDs) have the potential to greatly impact the surgical field by maintaining sterile conditions in healthcare environments. Google Glass (GG) and Microsoft HoloLens (MH) are examples of optical HMDs. In this comparative survey related to wearable augmented reality (AR) technology in the medical field, we examine the current developments in wearable AR technology, as well as the medical aspects, with a specific emphasis on smart glasses and HoloLens. The authors searched recent articles (between 2017 and 2022) in the PubMed, Web of Science, Scopus, and ScienceDirect databases and a total of 37 relevant studies were considered for this analysis. The selected studies were divided into two main groups; 15 of the studies (around 41%) focused on smart glasses (e.g., Google Glass) and 22 (59%) focused on Microsoft HoloLens. Google Glass was used in various surgical specialities and preoperative settings, namely dermatology visits and nursing skill training. Moreover, Microsoft HoloLens was used in telepresence applications and holographic navigation of shoulder and gait impairment rehabilitation, among others. However, some limitations were associated with their use, such as low battery life, limited memory size, and possible ocular pain. Promising results were obtained by different studies regarding the feasibility, usability, and acceptability of using both Google Glass and Microsoft HoloLens in patient-centric settings as well as medical education and training. Further work and development of rigorous research designs are required to evaluate the efficacy and cost-effectiveness of wearable AR devices in the future.

## 1. Introduction

Artificial intelligence (AI), particularly deep learning (DL), has facilitated the advancement of virtual reality (VR) and AR technologies. DL’s ability to perform object tracking and segmentation, as well as improve video resolutions, can reduce the computing power and costs needed for AR and VR systems and improve device performance. In the healthcare field, AR and VR technologies have been applied in a variety of areas, including laparoscopic surgery, robotic surgery, oral and maxillofacial surgery, guided biopsy, tumour resection, rehabilitation, cancer management, psychology, and neurosurgery. AR technology has been used to help surgeons inside the operating room (OR), as well as outside the OR for remote mentoring, patient education, resident training, and preoperative planning. It has also been used in orthopaedic procedures to assist surgeons in improving their speed and accuracy and has been applied in the treatment of spinal disorders. 

HMDs provide a hands-free display of information within the user’s visual field and have the potential to greatly impact the surgical field by maintaining sterile conditions in healthcare environments [1]. According to Rahman et al. [2], the use of this technology is rapidly expanding in the healthcare industry, and they predict that the market for HMDs in this sector will reach USD 5.1 billion within the next decade. 

Due to increasing demands for health sciences, new and effective options are required in this field. According to Moro et al. [3], VR and AR technologies are considered innovative learning tools that can promote hands-on learning experiences, for example, through the use of PlayStation and Google Glass [1,2]. VR uses a computer system/smartphone to provide a range of interactive digital experiences that mimic the real world [3]. It is based on an HMD and is associated with tactile and auditory sensations. In contrast, AR allows access to a real-world environment that is covered with interactive and digital elements and is frequently associated with a smartphone/tablet. This technology is not limited to gaming experiences but also has educational applications across a wide range of student stages. 

AR technology allows for three-dimensional knowledge of human organ systems and structures [3]. Google Glass is an optical HMD (similar to a pair of eyeglasses) that allows for the connection of a wearable 5.0-megapixel integrated camera and heads-up display to mobile phones via Wi-Fi. This project, which started in 2013, is capable of taking pictures with simple voice commands. Due to these characteristics, Google Glass is used frequently in surgical [2] and non-surgical settings [4]. According to recent studies, Google Glass is a good solution for people with colour blindness [5] and is useful for modulating gait in patients with Parkinson’s disease [6]. It is also used for playing recorded videos, transferring patient data for mentoring purposes, and addressing communication in a telemedicine context [7,8]. Moreover, Microsoft HoloLens was released in 2015 and is the first AR HMD capable of spatially capturing its environment. This technology is also used to facilitate and enhance remote medical training [7].

Although there are numerous advantages of using Google Glass in clinical settings, possible limitations are connected with its use among patients and healthcare professionals. More studies are required to better understand Google Glass’s data security, as well as its suitability in specialized medical applications. Another limitation of Google Glass is the lack of triage accuracy, which is necessary to identify and prioritize patients with the most urgent medical needs [7,8]. Moreover, the literature regarding Microsoft HoloLens is still scarce, mainly in terms of its use in the medical field. This technology is a non-occluding AR system with some disadvantages such as physical discomfort and pain, limited memory size, and lower resolution than full HD monitors. Additionally, at around 100 minutes, the battery life of Microsoft HoloLens is limited [7].

AR technology is widely used in healthcare settings to treat and help patients. Many studies have been reported in this field; however, they focus on a specific AR technology, for example, a focus on Google Glass in clinical and non-clinical settings [1,2,4]. In line with this, we plan to combine promising technologies, particularly Microsoft HoloLens and smart glasses.

In this systematic review related to the application of AR technology in the medical field, we will examine the current developments in wearable AR technology, as well as its medical aspects, with a specific emphasis on smart glasses and HoloLens. In line with this, we aim to identify the research gaps and problems regarding wearable AR technology in healthcare, as well as categorize the current research on wearable AR technology in this field. Furthermore, we would like to provide a road map of wearable AR-technology-related research in healthcare worldwide.

## 2. Materials and Methods

This manuscript partially followed the checklist and explanation for preferred reporting for systematic reviews and meta-analyses for scoping reviews (PRISMA-ScR) [9]. This manuscript has not been previously entered into databases such as PROSPERO.

### 2.1. Study Selection

In October 2022, we searched for recent articles published between 2017 and 2022 using the keywords “Head-Mounted Display”, “Head-mounted Device,” “Smart glasses”, “Google Glass”, and/or “Microsoft HoloLens” that were associated with applications in healthcare in the PubMed, Web of Science, Scopus, and ScienceDirect databases.

**Figure 1:**
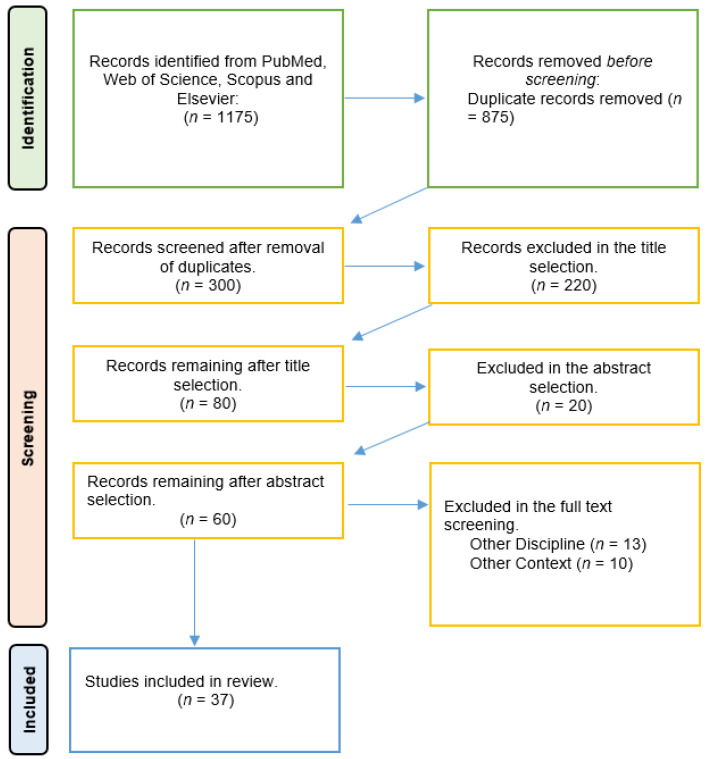
Flow chart of the selection process of the included studies.

A total of 1175 articles were gathered from the various databases. The authors screened around 300 original articles after removing duplicates. Upon analysis of the titles and abstracts, 60 full texts were considered relevant and underwent a detailed review, resulting in 37 manuscripts being included in the final analysis (see Figure 1). Articles on HMDs were categorized as smart glasses and HoloLens and were included in this review article. In instances where the full text of certain articles could not be obtained through conventional channels, efforts were made to reach out to the corresponding authors to obtain the necessary information. Despite these efforts, if the full text was not obtained after communicating with the corresponding authors, these articles were deemed ineligible for inclusion in our analysis.

### 2.2. Data Extraction and Analysis

The authors screened all articles individually, including a review of all full texts, and specific data were collected, namely authors’ names, publication year, country, sample size, study design/ study settings, AR technology used, medical speciality application, and categorization/classification of the literature. It is important to note that all data were analyzed quantitatively and qualitatively. 

The following were the inclusion criteria for this review: (1) original research, (2) involved the use of a head-mounted display (HMD) for a surgical task, (3) published in English, and (4) conducted in a clinical setting, either in real time or in a simulated environment.

## 3. Results

Out of the 1175 studies that were reviewed, 37 relevant review manuscripts were selected for further analysis and exploration. These 37 articles were divided into two main groups: 15 (approximately 41%) were conducted on smart glasses (usually using Google Glass) and 22 (approximately 59%) were conducted on HoloLens (usually Microsoft HoloLens). A summary of the 37 included studies is illustrated in both Table 1 (Google Glass studies) and Table 2 (Microsoft HoloLens studies).

### 3.1. Description of Included Studies Regarding Google Glass (or Other Similar Smart Glasses)

As discussed previously, Google Glass is a wearable technology in the form of eyeglasses and is associated with a high-definition camera that allows the user to interact using voice commands. Considering the studies based on Google Glass (or similar types of smart glasses), we selected 15 studies for extended analysis and incorporation into this review. It is important to note that all 15 studies were conducted in hospital settings (Table 1). Most of the selected studies were associated with the feasibility, safety, and efficacy of Google Glass in different medical settings. Although the majority of the smart glasses used in the selected studies were Google Glass, eight exceptions were considered. Harris et al. [10] chose ODG R-7 AR glasses with installed NuLoupes for their demonstration; Munusamy et al. [11], Kim et al. [12], and Sommer et al. [13] used Vuzix smart glasses in their research studies; Maruyama et al. [14] selected the Moverio BT-200 (Seiko Epson Corporation); Park et al. [15] and Jang et al. [16] selected the Moverio BT-35E smart glasses (Suwa, Japan: Epson Inc.); and the VR X-Ray glasses developed by Skilitics and Virtual Medical Coaching, New Zealand, were selected by Kato et al. [17].

**Figure 2:**
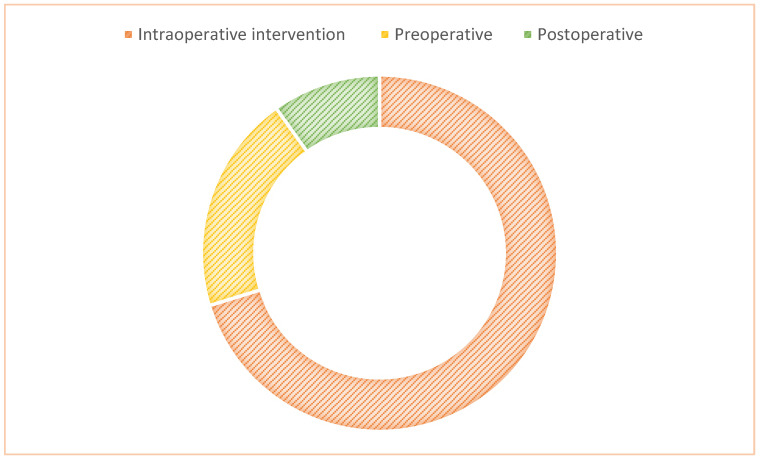
Use of Google Glass.

It is important to note that the vast majority of the studies examined the potential use of Google Glass as an intraoperative intervention (9/15, 60%) [10,11,13,14,15,18,19,20,21], as well as its potential use in preoperative/teaching (4/15, 26.7%) [12,16,17,22] and postoperative (1/15, 6.6%) [8] settings (Figure 2). Another analyzed study [23], which did not cover any of these three applications, was associated with Google Glass applications in the medical industry, as well as its useful contribution to physicians. 

**Figure 3:**
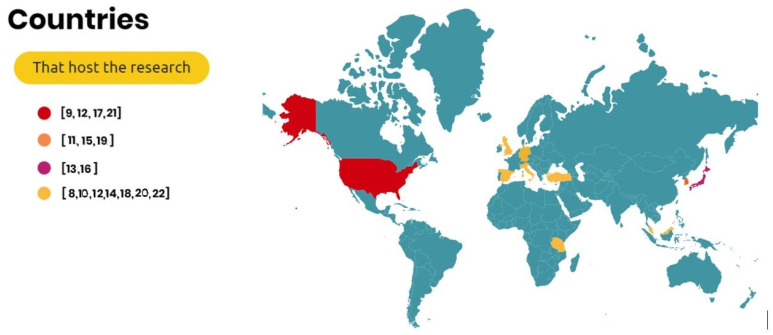
Locations of the studies focusing on Google Glass [8,9,10,11,12,13,14,15,16,17,18,19,20,21,22].

The authors selected international studies related to medical applications of Google Glass Figure 3 shows the countries that conducted the research. The studies selected were conducted in the USA [10,13,18,22], South Korea [12,16,20], Japan [14,17], Germany [21], Italy [8], the UK [15], Spain [19], Turkey [23], Malaysia [11], and Tanzania [13].

Regarding operative settings, Google Glass was used in various surgical specialities, including urological surgery [19,21], spinal surgery [13,20], oncological surgery [24], orthopaedic surgery [15], and neurological surgery [11,14,18] (Table 1). This technology was also applied in preoperative settings, namely dermatology visits [22] and nursing skills training [12]. According to recent research, AR smart glasses can also be used by non-surgical staff to control damage procedures [10]. Curiously, Piegari et al. [8] recently conducted a study on the application of Google Glass in veterinary forensic pathology. All the details are presented in Table 1. 

**Table 1:** The summary of the selected studies that focus on Google Glass applications in the healthcare field.

Ref	Purpose	Study Design/Setting	Sample Size	AR Tech Used	Medical Application
[12]	The goal of this study is to determine whether it is possible and effective to use telemedicine delivered through smart glasses to transmit video content during spine surgery.	During spine surgeries, a smart glasses system with an integrated camera and microphone was used to transmit intraoperative video for assistance.	3 patients of scoliosis correction surgeries	Vuzix Smart Glasses	Spine Surgery
[14]	This study aims to show the usefulness and advantages of using wireless smart glasses to improve ergonomics, and reduce disruptions during surgery.	The primary surgeon wore smart glasses during the procedure to enable heads-up visualisation of the intraoperative fluoroscopy.	A patient	Moverio BT-35E Smart Glasses (Suwa, Japan: Epson Inc.)	Orthopaedic Surgery
[13]	To evaluate the feasibility and accuracy of using smart glasses with augmented reality technology for neurosurgical navigation.	Two motion capture cameras were deployed to continuously track the location of the smart glasses in relation to the patient’s head (with brain tumours located in the brain surface).	2 patients with brain tumors	Smart Glasses (Moverio BT-200; Seiko Epson Corporation, Suwa, Japan)	Neurosurgery navigation
[17]	To determine the suitability of using Google Glass as a tool to improve the surgical training of neurosurgical residents.	Three cases were taken into consideration: (1) a minimally invasive lumbar diskectomy performed prior to surgery; (2) an emergent craniotomy recorded during surgery; and (3) the patient’s condition following a surgical mission to Mongolia.	N/A	Google Glass	Variety of clinical settings: Neurosurgery, and teaching tool
[8]	The purpose of this study is to determine whether Google Glass is a viable option for use in the field of veterinary forensic pathology.	On the basis of the animal’s outward appearance, its organs, and its anatomical characteristics, the images were gathered, sorted into three groups, and scored using a 5-point scale by five forensic pathologists.	44 forensic necropsies of 2 different species (22 dogs and 22 cats)	Google glass	Veterinary Forensic Pathology
[21]	To investigate patients’ perceptions of having a remote medical scribe present during office visits using Google Glass.	Participants filled out a 12-item survey and supplied demographic information. Descriptive and inferential statistics were used to evaluate the results.	170 patients were recruited from an outpatient dermatology clinic	Google Glass	Outpatient dermatology visits
[11]	To create a smart glass-based nursing skills training program and assess its usefulness and practicality for self-practice.	Before and after the intervention, the number of practise sessions was recorded, and perceived proficiency in fundamental nursing tasks was assessed.	30 undergraduate nursing students	Vuzix Smart Glass	Nursing Skill Training
[18]	Explore the potential benefits of using smart glasses in the surgery room and outpatient care settings in urology.	Eighty urologists were encouraged to utilise Google Glass in their daily surgical procedures and to share their experiences with other urologists. The assessment utilised a 10-point scale.	80 urologists	Google Glass	Urological surgery
[10]	To determine whether telemedicine delivered through smart glasses was a feasible and effective way to conduct ward rounds on neurocritical care patients during the COVID-19 pandemic.	Consecutive virtual and in-person ward rounds on neurocritical patients were performed by a random pairing of neurosurgery residents and specialists.	3 residents and 2 specialists	Vuzix M400 Smart Glasses	Neurosurgery
[22]	This study aims to examine the use of augmented reality smart glasses by physicians and their adoption of these products in the Turkish medical industry.	The Davis Technology Acceptance Model as a basis for a hypothesising framework. Exogenous elements were defined through a combination of semi-structured in-depth interviews, an expert panel.	71 out of 75 participants were used in the hypotheses testing.	Google Glass	ARSGs are not developed for task- or job-specific domains
[9]	To prove that a non-surgeon could follow a damage control procedure with the help of a wearable AR telescoping device.	A surgeon at a different location used a stand-alone, low-profile, commercially available wearable AR display to guide a nonsurgeon through proximal control of the distal external iliac artery on a surgical manikin at the same time.	The manikin wound pattern simulation—Testing.	Vuzix Smart Glasses	on-visual-axis telestration system
[16]	Evaluate skills and proficiency of medical staff when using VR (through HMD) compared to real-world radiographic training techniques.	Students are divided into: HMD-VRC (smart glasses) group and RP group (real physical equipment), then trained and their proficiency was evaluated. HMD-VRC group showed significant decrease in proficiency in skills related to palpation and patient interaction.	30 first-year radiology students	VR X-Ray (Skilitics and Virtual Medical Coaching, New Zealand)	Radiography education
[15]	Investigate the use of smart glasses for radial artery catheterization in infants’ patients.	The E-CUBE i7 machine was connected to the BT-35E smart glasses, which served as the HMD and provided a simultaneous display of the ultrasound screen.	116 patients, age less than 2 years	binocular Moverio BT-35E Smart Glasses &	Pediatric—Radiology

Based on our review of the literature, it is evident that Google Glass is a valuable tool for medical and educational applications [1,17,18,20,22]. Among its main advantages, this technology is easy to use, comfortable to wear, and has low distractibility, making it suitable not only for intraoperative interventions (surgeries) but also for diagnosis and as a learning tool.

### 3.2. Description of Included Studies Regarding Microsoft HoloLens 

The Microsoft HoloLens is based on AR technology and uses multiple sensors, advanced optics, and holograms that allow for the simulation of a VR world. It is considered a novel AR tool with multiple clinical and non-clinical applications in pathology. According to Hanna et al. [25], this device is comfortable to wear, easy to use, and provides sufficient computing power and high-resolution imaging. 

**Figure 4:**
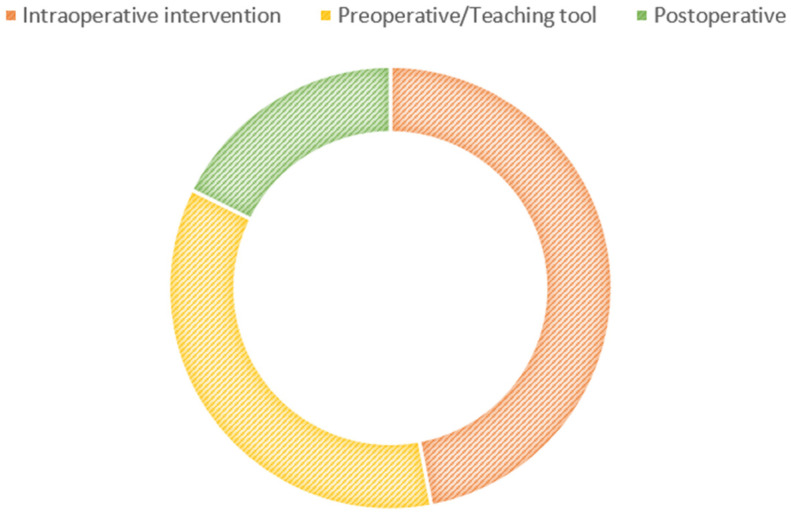
Use of Microsoft HoloLens.

A total of 22 studies that focused on Microsoft HoloLens matched our criteria and were selected for inclusion in this review. The data from these studies were analyzed and are discussed in this section. Similar to Google Glass, the studies based on Microsoft HoloLens were conducted in medical settings (Figure 4). 

The vast majority of the selected studies were associated with the potential use of Microsoft HoloLens as an intraoperative intervention (11/22, 50%) [25,26,27,28,29,30,31,32,33,34,35] and as a preoperative/teaching tool (7/22, 31.8%) [3,7,36,37,38,39,40], with a smaller number of studies investigating its use in post-operative (3/22, 13.6%) [41,42,43] settings and for measuring healthy adults [44] (Table 2). 

**Figure 5:**
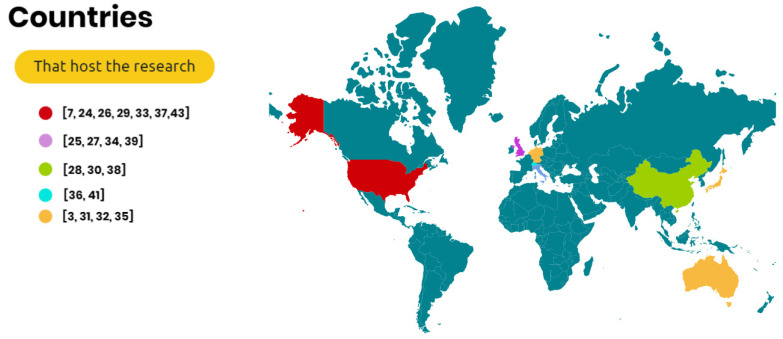
Locations of the studies focusing on Microsoft HoloLens [3,7,24,25,26,27,28,29,30,31,32,33,34,35,36,37,38,39,41,43].

The authors selected international studies focusing on medical applications of Microsoft HoloLens. Figure 5 shows the countries that conducted the research. A total of 31.8% of the selected studies were conducted in the USA [7,25,27,30,34,38,44], followed by the UK [26,28,35,40] (18.1%) and China [29,31,39] (13.6%). Other studies were conducted in Italy [37,42], Switzerland [41,43], the Netherlands [36], Germany [32], Japan [33], and Australia [3].

Regarding operative settings, Microsoft HoloLens was used in various surgical specialities, including anatomy pathology [25], otolaryngology surgery (head and neck) [27], cholangiography [22], and urological surgery [34], as well as in localization of perforated vessels/vascular localization system [26,39], for digital rectal examinations [28], in surgical 3D navigation [29], in shoulder arthroplasty [30], and in image-guided interventions [32] (Table 2). Moreover, this technology can be used in telepresence applications [39] or holographic navigation [36], general anatomy and physiology [38], forensic pathology [41], and shoulder and gait impairment rehabilitation [42,43]. Furthermore, Liu et al. [31] considered the use of Microsoft HoloLens in medical training and telementoring surgery based on a 3D point-tracking module. Another interesting aspect of Microsoft HoloLens is its ability to measure gait performance in healthy adults [44]. All details are summarized in Table 2. 

According to recent reports, Microsoft HoloLens is a useful tool for basic life support and defibrillation training [37], as well as medical and health sciences education [3]. Among its main advantages is that it is a hands-free technology and presents excellent hologram resolution and spatial sound. Microsoft HoloLens can help businesses with updates and make medical surgeries/diagnoses more effective, making Microsoft HoloLens suitable for use worldwide in a variety of useful applications.

**Table 2:** The summary of the selected studies that focus on Microsoft HoloLens for surgical medical intervention.

Ref	Purpose	Study Design/Setting	Sample Size	AR Tech. Used	Medical Application
[33]	Investigated the utility of intraoperative 3D holographic cholangiography.	In a hybrid operating room, 3D cholangiography was carried out during surgery. Using the data from the cholangiography, 3D polygon data were entered into the HMD.	2 patients	Microsoft HoloLens	Intraoperative Cholangiography
[35]	Introduced a flexible, device agnostic and precise HMD-based augmented reality framework for markerless orthopaedic navigation.	Demonstrated the concept. On a platform with Microsoft HoloLens 1, a markerless surgical navigation system to help with femoral bone drilling was built.	N/A	Microsoft HoloLens	Orthopaedic Surgery
[34]	Showed the feasibility of XRAS in penile surgery by presenting the first example of Microsoft HoloLens-assisted sophisticated penile revision surgery.	Incorporated common elements of the surgical process and the innovative XRAS technology superimposed a computer-generated image of the physician’s field. OHMD was used to create an extended reality (XR) interface.	N/A	OHMD, Microsoft HoloLens	Urological surgery
Wang et al. [7]	Created a new telepresence application utilising augmented reality.	Design of prototypes: gyroscope-controlled probe, video conferencing, and AR tied to VR.	N/A	Microsoft HoloLens	Development of one of the first telemedicine mentoring systems using Microsoft HoloLens
Hanna et al. [25]	Examined the use of Microsoft HoloLens in clinical and non-clinical pathological applications.	Virtual autopsy annotation, 3D gross and microscopic pathology specimen viewing, entire slide image navigation, telepathology, and real-time pathology–radiology correlation.	N/A	Microsoft HoloLens	Autopsy, gross and microscopic examination (anatomic pathology)
Pratt et al. [26]	Examined whether AR is useful for reconstructive surgery, with the precise diagnosis, dissection, and application of vascular pedunculated flaps.	AR overlay and comparison to the positions found by audible Doppler ultrasound were used to find vascular perforations.	6 patients with different clinical cases	Microsoft HoloLens	Localization of perforating vessels
Affolter et al. [41]	Identified the limitations of existing methods for showing medical image data during autopsies.	The presented method leveraged augmented reality to display basic DICOM image stacks.	Software and hardware	Microsoft HoloLens	Forensic autopsy (first test)
[40]	Examined the viability of delivering remote bedside instruction using a mixed-reality headset.	Senior physicians wearing HoloLens glasses led two MR sessions. The headset made it possible for the trainer and the medical students to communicate audiovisually in both directions.	24 patients, and 2 MR sessions	Microsoft HoloLens	Remote Bedside Teaching
van Doormaal et al. [36]	Examined the feasibility and precision of holographic neuronavigation using smart glasses.	Neuronavigation system programming on HoloLens for use in the operating room.	3 patients	Microsoft HoloLens	Holographic navigation
Rose et al. [27]	Designed a head-mounted augmented reality system for pinpointing the intraoperative localization of disease and normal anatomic landmarks in patients undergoing open head and neck surgery.	The use of computed tomography images to generate 3D digital models led to the formulation of a standard procedure.	N/A	Microsoft HoloLens	Otolaryngology—Head and Neck Surgery
Chen et al. [38]	Enhanced memory retention in anatomy and physiology.	Participants were tested through anatomy and brain physiology memory exams.	22 undergraduate students	Microsoft HoloLens	Anatomy and physiology
Condino et al. [42]	Explored shoulder rehabilitation using Microsoft HoloLens and real-time markerless hand tracking.	Analysis of traditional rehab. exercises to make sure the user was as comfortable as possible during the AR rehab. session	N/A	Microsoft HoloLens	Shoulder Rehabilitation (first wearable AR application)
Ingrassia et al. [37]	Examined the feasibility and acceptance of Holo-BLSD (the authors’ AR prototype) as a tool for basic life support training.	Participants utilised natural body movements and verbal commands to complete 3D technology-related activities. In addition, they completed a survey.	36 participants	Microsoft HoloLens	Basic Life Support and Defibrillation Training
Held et al. [43]	Examined the modulation of the gait pattern of stroke survivors during overground walking based on AV versus walking without AR performance feedback; investigated the usability of the AR system.	Development of a HoloLens-based system. Evaluation of gait movement kinematics, as well as the system’s usefulness and safety.	A patient	Microsoft HoloLens	Rehabilitation of Gait Impairments
Wenhao Gu [30]	Examined the use of Microsoft HoloLens to guide glenoid drilling during total shoulder arthroplasty, as well as the design and viability of a markerless image-based registration pipeline utilising Microsoft HoloLens and its built-in sensors.	A 3D image of the exposed glenoid surface was taken prior to surgery, both with and without occlusion.	A patient	Microsoft HoloLens	Shoulder arthroplasty
Jiang et al. [39]	Assessed the accuracy of a Microsoft HoloLens-based vascular localization system as the most crucial performance indicator of a novel localization system.	Using a 3D-printed model, the accuracy of a HoloLens-based vascular localization system was evaluated in a simulated operating room under varying settings.	N/A	Microsoft HoloLens	Vascular Localization System
Moro et al. [3]	Utilized Microsoft HoloLens or a portable tablet to evaluate the learning process.	Pre- and post-intervention assessments were provided to participants to gauge their information retention, and they were also required to respond to a questionnaire to gauge any negative health consequences, as well as how they felt about the module.	40 students (Between 17 and 25 years)	Microsoft HoloLens	Medical and health sciences education
Liu et al. [31]	Described a novel augmented reality system for telementoring surgery that combined a Microsoft HoloLens device with a three-dimensional (3D) point-tracking module.	A virtual surgical scene with pre-recorded surgical annotations was superimposed on the actual surgical scene, allowing the surgical trainee to operate in accordance with virtual instructions.	Experimental setup	Microsoft HoloLens	Medical training and telementoring surgery
Koop et al. [44]	Aimed to determine the accuracy of Microsoft HoloLens relative to three-dimensional motion capture (MoCap) in quantifying gait.	Statistical equivalency study utilising a five percent a priori criterion confirmed that biomechanical measurements acquired from the HoloLens device were equivalent to those acquired using MoCap.	10 healthy adults completed 9 walking trials	Microsoft HoloLens	Medical education and visualization of surgical procedures
Rüger et al. [32]	Aimed to better comprehend the advantages and limits of this technology for ultrasound-guided therapies.	Utilized a combination of approaches, including a randomised crossover trial and a qualitative investigation.	Participants (n = 20)	Microsoft Hololens)	Needle placement and ultrasound

## 4. Discussion

### 4.1. Comparison of Google Glass and Microsoft HoloLens: Strengths and Limitations

The use of AR technology for clinical and non-clinical applications is promising and has attracted the attention of consumers and corporations. In the beginning, these technologies were commonly used in gaming, personal entertainment, and various business applications. In practice, the use of advanced technology and AR has increased in medical applications [23]. 

To the best of our knowledge, only a few international literature reviews have been reported to date [1,2,4,23]. This work is a complete systematic review of Google Glass and Microsoft HoloLens studies (n = 37), with a specific emphasis on their various applications in medical settings. Importantly, we focused on data on surgical settings. In this systematic review, we analyzed recent clinical studies and pilot investigations based on Microsoft HoloLens and smart glasses in medical settings. The feasibility, acceptability, and possible applications of these devices were explored. 

### 4.2. Comparison of Google Glass and Microsoft HoloLens: Strengths

Medical education, health training, different surgical fields, pathology, and autopsy are considered some of the main applications associated with Google Glass and Microsoft HoloLens. Among the main advantages, they can virtualize online information without interruption while saving time. Moreover, video capture can be used in the education of medical students, as well as for the publication of articles [23]. AR technology is particularly useful during surgical interventions that take place in a confined space or near delicate anatomical structures [7]. Importantly, these headsets can respond to users’ voices, hands, and eyes.

Hanna et al. [25] reported on the usefulness of Microsoft HoloLens for autopsies, gross and microscopic examinations, and digital pathology. HoloLens technology is not associated with VR nausea or 3D headaches, making it comfortable for users. This device also allows for the projection of 3D images onto objects. Other applications include remote supervision and annotation, 3D image viewing and manipulation, telepathology, and real-time pathology–radiology correlation. HoloLens can provide vital information to surgeons, such as the location of cancerous tissue [7]. Another strength of these headsets is their usefulness in telemedicine platforms.

In contrast with Microsoft HoloLens, Google Glass is small and has an unobtrusive design; it can be used throughout the day while still being considered a fashion object. Google Glass has the potential to address communication and educational challenges in a telemedicine context [7]. Recently, Munusamy et al. [11] developed research based on smart glasses in relation to the COVID-19 pandemic. The authors concluded that the use of smart glasses with neurosurgical patients in critical care was feasible, effective, and widely accepted as an alternative to physical ward rounds during the coronavirus disease 2019 pandemic.

Furthermore, in metaverse education and training, augmented reality can be used in medical education to create holographic museums and virtual rooms for ophthalmic teaching, anatomy instruction, and surgical simulations [45]. The use of virtual reality can standardize education and reduce discrepancies in the education of medical students. Additionally, the authors of [46] mentioned online platforms as a cheap and feasible way to educate people through distance learning programs organized by experts, universities, or governments. This method allows for 24-h access and indefinite registration, with practical elements included in the programs.

### 4.3. Comparison of Google Glass and Microsoft HoloLens: Limitations

However, these technologies are associated with some limitations. In contrast with Microsoft HoloLens, Google Glass is associated with an information-only display that appears on one side. Moreover, a study showed that Google Glass was unable to capture all relevant anatomy during a specific surgery, and it also has short battery life, video recording time limits, display overexposure, and small screen size. Google Glass is considered an expensive device, starting at around USD 1500. Moreover, no increase in disaster telemedicine triage accuracy was found [7]. Another important aspect is the requirement of a good Internet connection, mainly in surgeries, to avoid connection interruptions and time lags in communication [4,23,41]. According to Basoglu et al. [23], smart glasses based on AR technology cannot capture every minute and specific detail. 

Another study that aimed to compare the precision and user preferences of different AR methods for HMDs, including Google Glass [47], confirmed the screen position problems of Google Glass. The results showed that most users preferred the camera-user perspective (CUPR) over the user perspective (UPR), due to the small size of the Google Glass display and the tedious calibration process. However, tracking jitter was more noticeable in both UPR and CUPR, causing disturbances to users while placing virtual objects in the correct positions. The users also pointed out that the delay in virtual information superposition affected the visual coherence of AR on HMDs. The method was improved by developing the visual tracking and user’s field of view, as well as by tackling the delay in the exhibition of the virtual elements.

Theoretically, Google Glass is voice-operated; however, many repetitions (3–5 times) are required in order to recognize and analyze a voice. In line with this, some changes must be considered before its integration into the surgical field. Although it can be considered a useful supplement to traditional monitors, it is not recommended to be used as an independent monitor [1]. According to a recent review conducted by Dougherty and Badawy [4], participants were not satisfied with Google Glass’s battery life, as well as its poor camera quality and potential to infringe on patient privacy. More improvements and developments in its data security and specialized medical applications are required [7].

On the other hand, Microsoft HoloLens’s memory size and battery life are not among its advantages (approximately 100 minutes when running an application before having to be charged again) [7]. In contrast with Google Glass, Microsoft HoloLens is more complex and associated with immersive computing tasks. Microsoft HoloLens has a significantly lower resolution and its weight is another disadvantage, which has been associated with discomfort and eye pain. It is also more expensive than Google Glass, with costs starting at around USD 4000. Another important limitation of Microsoft HoloLens is that it can only be used indoors and in closed environments [7]. Moreover, further testing is required to validate Microsoft HoloLens for use in routine clinical practice. Microsoft HoloLens needs to be further explored and investigated as an effective telemedicine AR device.

### 4.4. Strengths of this Study

To the best of our knowledge, this is one of the most comprehensive SRs that covers and evaluates a vast majority of the wearable AR technology used in the medical field, whereas most previous SRs focused on one specific aspect. Moreover, this work provides a road map for researchers and policy makers for the use of AR technology in healthcare. The authors aimed to categorize all the literary works and synthesize their outcomes. The initial strategy guidelines and recommendations for the established systematic review methodology were followed as expected by the authors. 

### 4.5. Limitations of the Study

Despite the overall promising data regarding the feasibility and the acceptability of using AR technology (mainly Google Glass and Microsoft HoloLens) in different surgical settings, there are some possible limitations associated with their use [1,2]. So, the potential methodological limitations of our systematic review should be discussed. We should also consider that some of the selected studies reported in Section 3 included a relatively small sample size. Our findings were corroborated by a recently published study [3], which was also characterized by a generalization of the results due to the small sample size. Furthermore, a review article is limited compared to an original research article; a possible risk of bias can be present where only the positive study results are considered. 

Although the authors extensively search for studies on AR applications in the medical field, there is a possibility that a few articles were missed during the literature review search. The authors of this review did not have full access to some of the articles, which may be another limitation. It is important to note that the exclusion of conference proceedings may have affected the quality of this review. Further investigations with rigorous research designs are required to evaluate the efficacy and cost-effectiveness of AR devices in the future. The implementation of and investment in numerous new healthcare technologies are required. Furthermore, clinicians may be better able to understand the best devices to use with their patients. However, in general, the use of both Microsoft HoloLens and Google Glass devices can be considered of great benefit to the use of wearable devices in medicine.

## 5. Conclusions

This article discusses the potential impact of head-mounted displays, specifically Google Glass and Microsoft HoloLens, on the healthcare industry as wearable augmented reality devices. The authors conducted a comparative survey of 37 recent studies that examined the use of these devices in various medical applications, such as surgical procedures, pre-operative care, medical training, and rehabilitation. The results of these studies suggest that both Google Glass and Microsoft HoloLens have potential uses in healthcare settings and they have received positive feedback in terms of their feasibility, usability, and acceptability. 

Several studies have yielded promising results regarding the feasibility, usability, and acceptability of using Google Glass or Microsoft HoloLens in patient-centred or student training settings. Regarding the articles analyzed, we can consider these devices as interesting tools that could help to improve the quality of patient care. Even with their technical limitations, the use of these technologies is widely reported due to their potential for use in surgical settings, as well as non-clinical fields.

For users to select a suitable device, both Google Glass and Microsoft HoloLens have strengths and limitations as wearable augmented reality devices in the healthcare sector. Google Glass offers a compact design, low cost, and the ability to respond to voice commands, making it ideal for telemedicine communication and education. However, it has limitations such as short battery life, limited video recording time, a small display, and the need for a good Internet connection. On the other hand, Microsoft HoloLens offers high-quality 3D images and a comfortable user experience without VR nausea or headaches. It is useful for autopsies, telemedicine, and surgery, but it is complex, heavy, and expensive, and has a narrow field of view, making it less suitable for outdoor use.

Based on a comparison of the two devices, the best device depends on the intended application and the specific needs of the user. If the user is looking for a more cost-effective device for telemedicine communication and education, Google Glass might be the better choice. However, if the user needs a device for surgery or autopsies, Microsoft HoloLens might be more suitable due to its 3D imaging capabilities and comfortable wearability. In any case, it is important to consider the limitations and trade-offs associated with each device before making a final decision.

However, the authors also note some general limitations, such as low battery life and limited memory size, and recommend further research and development to fully evaluate the efficacy and cost-effectiveness of these wearable augmented reality devices. Further studies with rigorous research designs are required to evaluate the efficacy and cost-effectiveness of AR devices. The use of both Microsoft HoloLens and mobile-based AR devices in medical settings can be considered beneficial.

## Data Availability

Data are available from the corresponding author upon request.
